# In-depth tube assessment by ultrasound: double twist sign to evaluate endotracheal tube depth—results of a cadaver diagnostic accuracy study

**DOI:** 10.1186/s40635-026-00937-x

**Published:** 2026-07-17

**Authors:** Jorge Ernesto Lopez Matta, Marissa Ginette Vive, Carlos Vsevolod Elzo Kraemer, Jeroen Alphons Janson, Marco Cornelis de Ruiter, Pieter Roel Tuinman, Robbert Godfried Notenboom, David Johannes van Westerloo

**Affiliations:** 1 Department of Intensive Care Medicine, Leiden University Medical Centre, Leiden, The Netherlands; 2 Department of Intensive Care, Amsterdam University Medical Center, Amsterdam, The Netherlands; 3 Amsterdam Leiden Intensive care Focused Echography (ALIFE, www.alifeofpocus.com), Amsterdam-Leiden, The Netherlands; 4 Department of Anatomy and Embryology, Leiden University Medical Center, Leiden, The Netherlands

**Keywords:** Endotracheal intubation, Tube depth, Double twist sign, Point-of-care ultrasound, Airway management, Intensive care

## Abstract

**Purpose:**

An adequate endotracheal tube depth is crucial for airway management and effective mechanical ventilation, as both too deep and superficial tube placement can be harmful to the patient. The aim of this study was to evaluate endotracheal tube depth using the ultrasound double twist sign as a new diagnostic tool using a cadaver model.

**Methods:**

In this diagnostic accuracy study, ultrasounds were performed by 12 sonographers (2 expert and 10 novice users, consisting of residents and specialists) on 3 cadavers. Novice users learned the technique in 15 min. Tubes were randomized to be placed in adequate, deep (tube tip touching main carina) or superficial (cranial edge of cuff touching vocal cords) position.

**Results:**

The double twist sign showed an overall success rate of 90.6% to identify tube depth. It was particular reliable in identifying and ruling out deep placement, with a sensitivity of 99.2%, a specificity of 98.5%. The mean time to identify deep placement was 6.4 s; with an overall mean assessment time of 18.4 s. Test performance was not significantly impacted by cadaver (BMI, neck circumference) or sonographer characteristics ( sonographer experience); however, the study was not powered to exclude clinically meaningful effects of anatomy.

**Conclusion:**

In an embalmed cadaver model, the double twist sign enabled rapid and accurate assessment of endotracheal tube depth using POCUS( Point of care ultrasound)with an air-filled cuff. These findings support feasibility and diagnostic potential, but clinical performance in ventilated patients and comparison against radiography require prospective validation.

**Supplementary Information:**

The online version contains supplementary material available at 10.1186/s40635-026-00937-x.

## Background

Correct endotracheal tube depth is crucial for secure airway management and effective mechanical ventilation. Although the incidence of tube malpositions varies largely among populations and settings, incorrect tube depth (esophageal, superficial and deep/endobronchial) and associated complications occur in up to 25% of intubations in high risk populations) [1]. Superficial tube placement has been observed in 8% of prehospital intubations [1], while mainstem intubations occurred in 4.2% of all intensive care unit (ICU) intubations [2] and 1.2% of emergency room intubations [3]. In specific populations, such as obese patients, incorrect tube depth was observed in 15% in an elective setting [4]. Both superficial and deep tube placement can be harmful and even detrimental to the patient. Superficial positioning may cause vocal cord or recurrent laryngeal nerve injury and accidental tube luxation [5]. Deep or endobronchial tube placement can result in hypoventilation of the non-ventilated lung and hyperventilation of the intubated lung, potentially causing hypoxemia, atelectasis, and barotrauma, potentially culminating in pneumothorax [6].

Several clinical clues and tools are commonly used to estimate endotracheal tube depth. Clinical evaluation using tube markings and chest auscultation is notably unreliable [7]. Although chest X-rays and capnography provide a more reliable answer, these techniques also have important limitations. Waiting for a chest X-ray may delay recognition of inadequate placement, during which the patient’s condition may deteriorate. In addition, chest X-rays are often unavailable in the operating room or prehospital setting and exposes the patient to radiation. Capnography is not always available and has limited utility in detecting superficial positioning. It is also unreliable in specific situations involving low carbon dioxide production, such as during cardiac arrest [8, 9].

Point of care ultrasound (POCUS) has been proposed as a solution to overcome these limitations as an adjunct to standard care because of its rapid, low-cost and non-invasive nature, as well as its applicability across wide range of clinical settings [9]. It has already been shown to reliably discriminate between endotracheal and esophageal intubation [10]. Several ultrasound techniques have been suggested to identify tube depth, relying on direct or indirect detection of the tube. A frequently used indirect approach is to check for bilateral lung sliding [2, 11]. However, this does not discriminate between endobronchial intubation or other pathology such as pneumothorax. In the emergency room, only 57% of patients that showed unilateral lung sliding after intubation were shown to have endobronchial tube placement, with the remainder being attributed to pneumothorax or chronic underlying lung disease [11].

Visualization of the tube within the trachea with POCUS is more challenging, as ultrasound waves are reflected back by the air between the trachea mucosa and tube. Extensive work by Gottlieb and others showed that cuff visualization becomes possible when the cuff is filled with saline instead of air [9, 12–16]. Although saline inflation may facilitate cuff visualization in research settings, it is not part of routine airway management and may be discouraged for many air-cuff tubes according to manufacturer guidance. Therefore, techniques that perform with standard air-inflated cuffs may be easier to translate into routine care [17, 18].

Therefore, we developed an ultrasound technique to reliably determine the depth of the endotracheal tube using an air-inflated cuff. The ‘twist sign,’ described by Gottlieb and colleagues, refers to a visible rotational movement of peri-tracheal tissues on transverse neck ultrasound when the endotracheal tube is deliberately rotated in the mouth. This dynamic maneuver can enhance recognition of tracheal versus esophageal tube position by producing a characteristic motion pattern at the level of scanning [19, 20],

We extended this concept by applying the twist maneuver at two anatomical landmarks “double twist sign” —the suprasternal notch and the cricothyroid membrane—to infer tube depth (deep/adequate/superficial) based on whether a twist signal is present at each level.

The primary aim of this study was to evaluate the diagnostic accuracy of the double twist sign to evaluate endotracheal tube depth. Secondary aims included evaluating the time needed to identify tube depth and to compare test performance among subgroups of sonographers.

## Methods

### Study design

This diagnostic accuracy study was conducted in the anatomic skills laboratory of Leiden University Medical Center (LUMC)between February and April 2025, using embalmed human Caucasian cadavers obtained from body donation. The goal was to evaluate tube depth using the double twist sign. For each ultrasound assessment, the endotracheal tube depth was randomized using Castor EDC (Amsterdam, The Netherlands). Intubation was performed by an experienced ICU physician (JLM), verifying tube depth using a bronchoscope (AMBU scope, Ballerup, Denmark) as reference test. Tubes size 7.0 were used for women and size 8.0 for men (Shiley tubes, Medtronic, USA). The cuff was inflated with 10 cc of air.

Tube positions were categorized as follows:

- Adequate: cranial edge of the cuff > 2 cm below the vocal cords and tube tip >3 cm above the main carina.
- Deep: tube tip touching main carina.
- Superficial: cranial edge of the cuff touching the vocal cords.

In the anatomic skills lab, the cadaver was placed in supine position on the dissection table. A curtain was placed across the cadaver’s chin, thereby dividing the room into two zones: an intubator area at the cranial end and an ultrasound area at the caudal end, thus blinding the sonographer from tube placement.

The diagnostic approach, the ‘double twist’, consisted of evaluating the twist sign at two anatomical landmarks: the suprasternal notch and cricothyroid membrane. After intubation with the cadaver’s head maintained in sniffing position, the sonographer was asked to evaluate tube depth using a handheld ultrasound device equipped with a linear probe (Phillips Lumify L12-4 linear array transducer). The sonographer positioned the linear probe in axial plane on the neck of the cadaver, starting at the suprasternal notch. The twist maneuver was then performed by the instructor, by moving the endotracheal tube from one mouth corner to the other (in between the lateral teeth line), creating a rotating movement of the tissue surrounding the cuff which is visualized by ultrasound. The twist sign was considered positive if a rotating motion of the tissue around the trachea was observed. Then the procedure was repeated with the probe positioned directly below the cricothyroid membrane. An adequately positioned tube corresponded with a positive twist sign on the suprasternal notch and negative twist sign on the cricothyroid membrane. If the test was positive in both positions, it was concluded that the tube was in superficial position. If the test was negative in the suprasternal notch, it was concluded that the tube was in deep position without performing the ultrasound on the cricothyroid membrane(Supplemenatal Video S1 and S2).

Time from start of the twist sign until localization was recorded. After the ultrasound examination, the intubator checked for potential displacement of the tube during the procedure. Then, to provide the sonographer with feedback and support learning, the sonographer was informed of the actual tube position.

### Cadaver model

For the study, three cadavers (2 female, 1 male; mean age ± SD, 87.0 ± 4.0 years; range, 83–91 years) of varying body composition were selected from eight available specimens: Cadaver A (female, body mass index [BMI] 22 kg/m^2^, neck circumference 38 cm), B (female, BMI 22 kg/m^2^, neck circumference 47 cm) and C (male, BMI 26 kg/m^2^, neck circumference 56 cm) (Table S1). To be suitable for the study, cadavers needed sufficient jaw and neck flexibility to allow for intubation. Cadavers with neck trauma, subcutaneous emphysema or scars to the neck were not selected (Figure S1). Body donor consent was obtained from all donors through the institutional body donation program. Embalmment was within 36 h after death using the F4L method [21]. Fixation was by femoral artery perfusion with F4L-embalming fluid (Fix for Life BV, Leiden, The Netherlands) containing 0.2–0.3% formaldehyde per body weight. The cadavers were preserved in F4L-immersion fluid (Fix for Life BV) for at least one year. The F4L-embalming method [21] is a specific type of body preservation that accurately preserves anatomical/histological features and also guarantees high-quality ultrasound images [22]. In addition, F4L-embalmed cadavers have been successfully used as a suitable and realistic airway management training model for basic and advanced airway maneuvers [23, 24]. The authors state that every effort was made to follow all local and international ethical guidelines and laws that pertain to the use of human cadaveric donors in anatomic research [25]. Institutional review board approval was not required for this study.

### Participants

The double twist ultrasound technique was developed and evaluated by two ICU physicians, one intensivist (JLM) and one resident (MV). They were considered expert users(“Developers”) and sequentially performed 15 ultrasounds per cadaver (total *n* = 90). To improve generalizability, 10 other physicians (5 intensivists and 5 ICU residents) novice to this technique (novice users) with varying experience with ultrasound and demographics were asked to perform 10 ultrasounds per cadaver(total *n* = 300) (Table S2). After a standardized15 minute training session, the sonographers were taught to perform the technique using a flowchart, live demonstration and a brief supervised practice on cadaver A (for teaching material, see Figure S2). Consequently, novice users were novice to the double twist technique, but recorded scans reflect performance after an initial orientation rather than a true first-ever attempt. Sonographers started on cadaver A, then progressed to B and lastly C. In doing so we intended to expose sonographers to increasingly challenging sonographic views (representing clinical practice) and to evaluate whether the skills practiced on one cadaver, could also be applied on other cadavers with different anatomical characteristics. It was logistically not feasible to rotate cadavers after each ultrasound. Cumulative operator experience was grouped in five-scan bins (cumulative experience group: Developer, 1–5, 6–10, 11–15, 16–20, 21–25, 26–30). This yielded 50 observations per novice bin (10 novices × 5 scans), and 90 observations in the Developer group. Because cadavers were examined in a fixed order, learning and order effects may confound cadaver-specific differences.

### Sample size

We prespecified a precision target corresponding to a 95% confidence-interval half-width ≤ 10% for per-position diagnostic performance, assuming approximately balanced assignment across positions (one-third per position). To account for within-reader clustering, we applied a design effect and planned a total of 390 scan assessments. This sample size was chosen to achieve the desired precision for groupwise estimates and to support stable estimation in marginal and mixed-effects models (generalized estimating equations [GEE] and generalized linear mixed models [GLMM]) across experience strata.

### Statistical analysis

Analyses were performed in IBM SPSS v29 and R version (version 4.5.2; 2025-10-31; R Foundation for Statistical Computing)(geepack for generalized estimating equations [GEE], lme4 for mixed-effects models).

*Diagnostic performance*. Sensitivity, specificity, positive predictive value (PPV), negative predictive value (NPV), and overall success were estimated with generalized estimating equations (GEE; binomial logit), clustering on sonographer with an exchangeable working correlation and including cadaver as a fixed effect to adjust for systematic differences between the three cadavers. Cluster-robust (sandwich) two-sided 95% confidence intervals were reported; cadaver contributions were averaged with equal weights.

*Time-to-classification*. Time (seconds) to identify tube depth was summarized as mean with two-sided 95% confidence intervals calculated using the t-distribution based on the standard error of the mean. These confidence intervals are descriptive and do not account for clustering by sonographer or cadaver.

*Learning/experience*. Cumulative operator experience was grouped in five-scan bins (Developer; 1–5, 6–10, 11–15, 16–20, 21–25, 26–30). Learning curves were estimated using mixed-effects logistic regression (binomial logit) with crossed random intercepts for sonographer and cadaver to account for repeated assessments. For sensitivity curves (Fig. 2), models were fitted within each true tube-position stratum (deep or superficial), with the outcome defined as correct identification of that position (yes/no). For specificity curves (Figure S3), models were fitted within the complementary strata (non-deep or non-superficial), with the outcome defined as correct exclusion (yes/no). Plotted points represent marginal model-predicted probabilities per experience bin with two-sided Wald 95% confidence intervals. Linear trends across experience were assessed in mixed-effects models treating experience as an ordinal continuous predictor.

*Multivariable model*. A mixed-effects logistic model with crossed random intercepts for sonographer and cadaver included fixed effects for cumulative experience (five-scan bins), sonographer ultrasound experience (years), and cadaver characteristics (age, BMI and neck circumference). Neck length and tracheal length were excluded due to nearperfect collinearity in the three-cadaver sample, and study phase (early vs. late) was omitted to avoid collinearity with the learning-bin variable derived from the same scan count. Adjusted odds ratios with two-sided Wald 95% confidence intervals and p-values are reported. Random-effect variance components were estimated near zero (singular fit); given the small number of cadavers (*n* = 3), this suggests negligible residual between-cadaver variability after accounting for fixed effects, and the model effectively behaves as a fixed-effects logistic regression with respect to cadaver.

## Results

A total of 390 ultrasound assessments were performed by 12 sonographers. Tubes were randomized to the following positions: adequate (*n* = 133), deep (*n* = 132), and superficial (*n* = 125). The mean time needed to identify tube depth was 18.4 s (95% CI 16.8–19.7 s). Substantially less time was needed when the tube was placed deep, with a mean time of 6.4 s (95% CI 5.5–7.2 s), as compared to the adequate and superficial positions for which a mean of 24.8 s (95% CI 22.2–27.5 s) and 23.8 s (95% CI 21.4–26.2 s) were needed respectively.

Table 1 presents the cross-classification of ultrasound findings versus the bronchoscopic reference standard. Ultrasound classification matched the reference in 355 of 390 assessments (91%). As observed in Table 2; The cluster-adjusted overall success rate was 90.6% (95% CI 87.5–93.0). Sensitivity and specificity were 99.2% (95% CI, 95.2–99.9) and 98.5% (95% CI, 96.7–99.3) for deep placements, 82.1% (95% CI, 74.1–88.0) and 95.5% (95% CI, 93.5–96.9) for superficial placements, and 90.4% (95% CI, 85.2–93.9) and 92.0% (95% CI, 87.6–94.9)for adequate placements. Positive and negative predictive values are provided in Table 2.

**Table 1:** Confusion matrix of ultrasound classification versus true endotracheal tube depth (*N* = 390)

Ultrasound Classification	Adequate	Deep	Superficial	Total
**True Endotracheal Tube Depth (bronchoscopy)**				
Adequate	120 (90.2)	1 (0.8)	18 (14.4)	139
Deep	1 (0.8)	131 (99.2)	3 (2.4)	135
Superficial	12 (9.0)	0 (0.0)	104 (83.2)	116
Total	133 (100.0)	132 (100.0)	125 (100.0)	390

Values are n (%). Percentages are column percentages (within true tube depth category)

**Table 2:** Cluster-adjusted ultrasound diagnostic performance metrics by tube position

Tube position	Metric	Point estimate	95% CI
Adequate	Sensitivity	90.4%	85.2–93.9%
	Specificity	92.0%	87.6–94.9%
	Positive predictive value (PPV)	85.3%	78.6–90.1%
	Negative predictive value (NPV)	94.3%	91.7–96.2%
Deep	Sensitivity	99.2%	95.2–99.9%
	Specificity	98.5%	96.7–99.3%
	PPV	97.0%	93.6–98.5%
	NPV	99.6%	97.5–99.9%
Superficial	Sensitivity	82.1%	74.1–88.0%
	Specificity	95.5%	93.5–96.9%
	PPV	88.4%	70.2–96.1%
	NPV	92.5%	88.9–94.9%
Overall	Success rate	90.6%	87.5–93.0%

Generalized estimating equations (binomial, logit link; exchangeable correlation structure) were used with sonographer as the clustering variable and cadaver as a fixed effect

Values are point estimates with cluster-robust 95% confidence intervals (CI). PPV = positive predictive value; NPV = negative predictive value

Metrics are shown for each tube position (Adequate, Deep, Superficial)

The overall row reports the success rate for all classifications combined

**Fig. 1:**
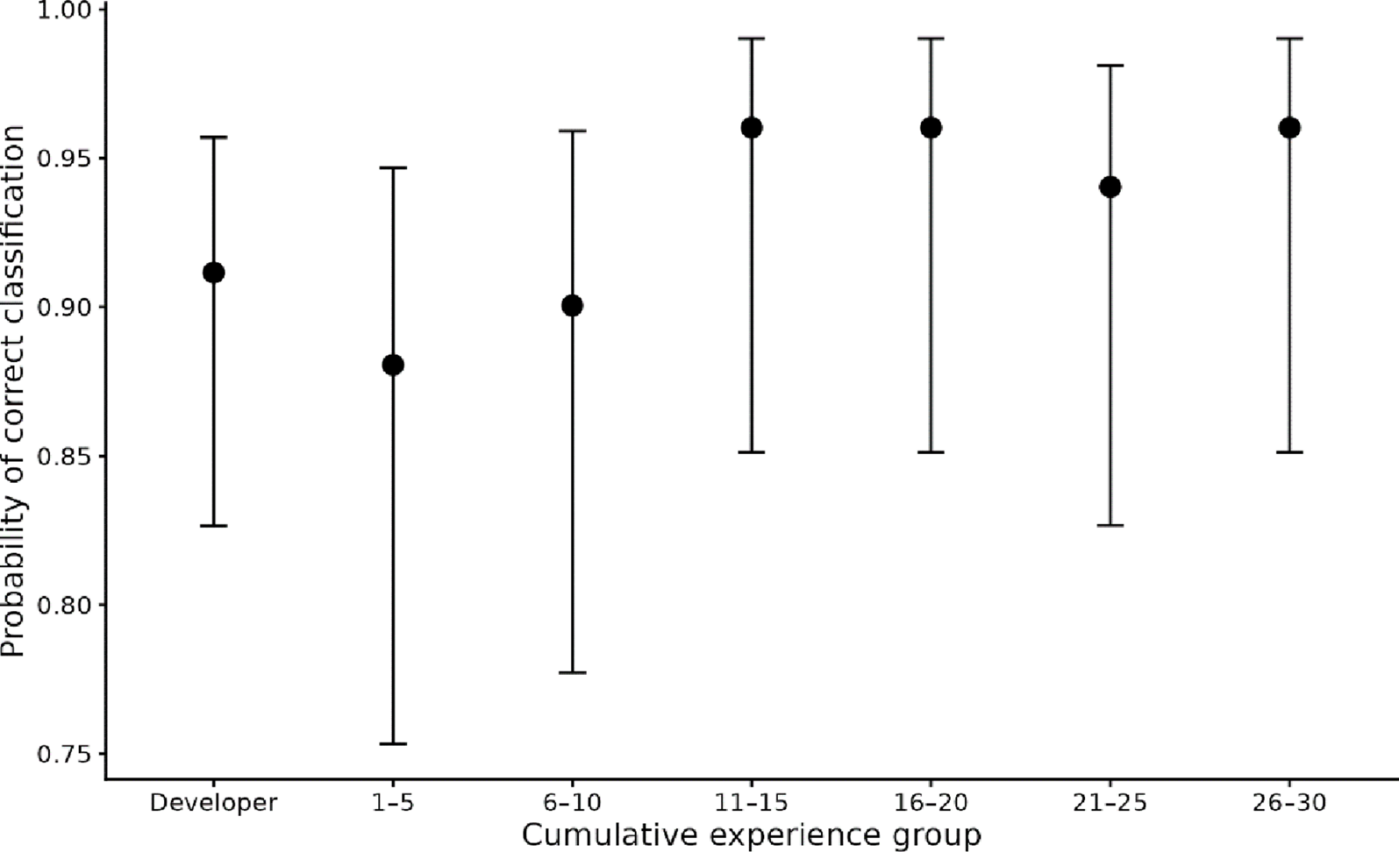
Probability of correct classification by cumulative experience group. Points show model-predicted probabilities from a generalized linear mixed model (binomial logit) with crossed random intercepts for sonographer and cadaver. Error bars indicate two-sided Wald 95% confidence intervals. Predicted probabilities remained high (≈0.88–0.96) across experience groups, indicating rapid attainment and maintenance of proficiency

Model-based probabilities of correct classification across cumulative experience groups are shown in Fig. 1.

**Fig. 2:**
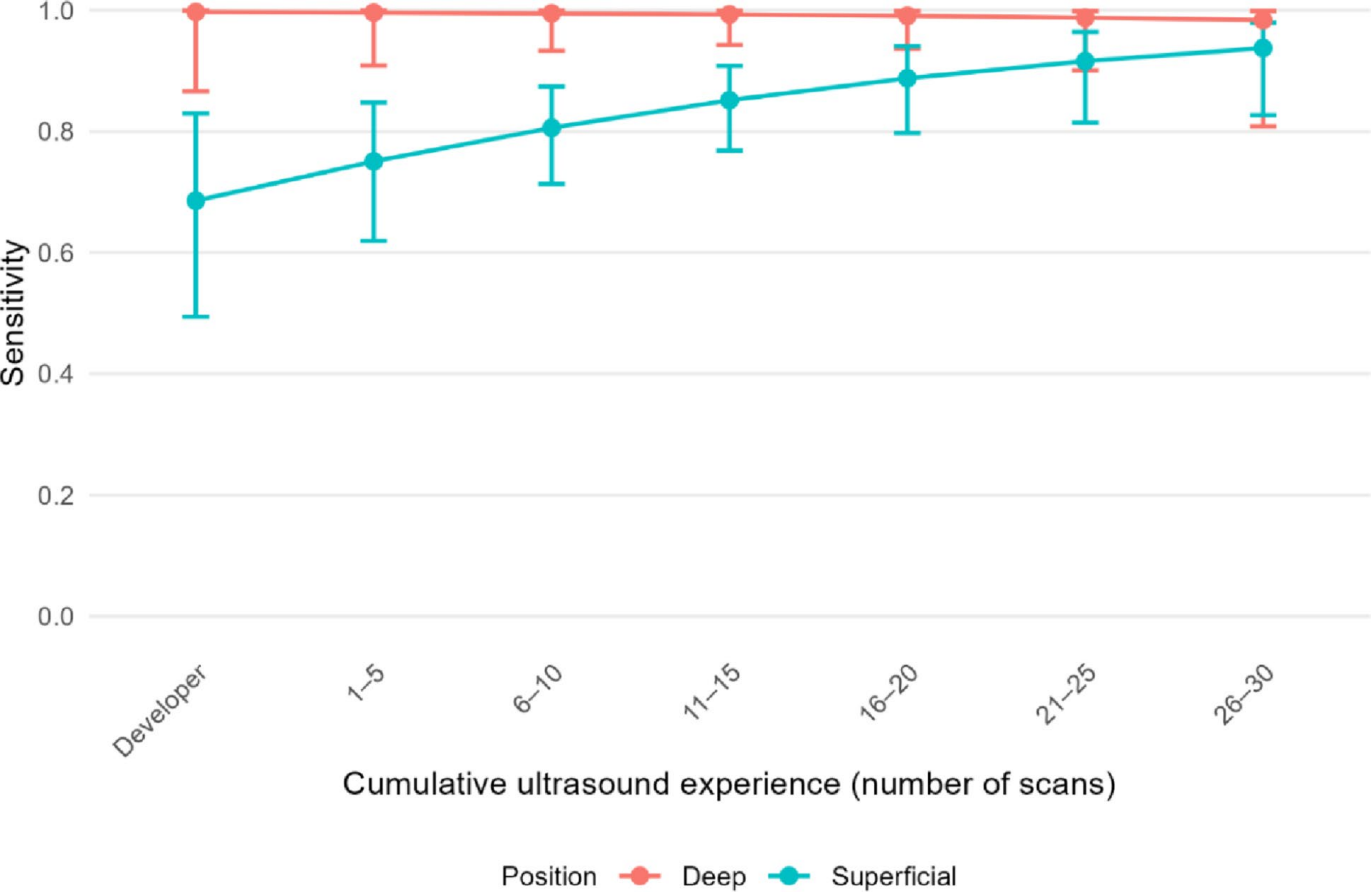
Clustered sensitivity learning curves for deep and superficial tube position detection by cumulative ultrasound experience. Sensitivity (points) with 95% confidence intervals (bars) for deep and superficial tube positions across cumulative ultrasound experience (grouped in blocks of 5 scans). Estimates are from a mixed‑effects logistic regression with random intercepts for sonographer and cadaver

Predicted probabilities ranged from approximately 0.88 to 0.96 with overlapping 95% confidence intervals, suggesting no consistent change in accuracy with increasing experience. Sensitivity by cumulative operator experience is shown in Fig. 2. Sensitivity for deep placements was high across all experience bins, whereas superficial placements started with lower sensitivity, increased in the early bins and then plateaued. In mixed-effects model treating cumulative experience as a linear predictor, these apparent changes were not statistically significant (p-for-trend = 0.51; interaction between position and experience *p* = 0.19). Specificity remained high and stable across all experience groups for both deep and superficial positions. In mixed effect models including cumulative experience as a linear predictor, we did not find a statistically significant linear trend in specificity with increasing experience (p-for-trend = 0.22), and the trend did not differ between deep and superficial placements(interaction *p* = 0.45).

Results of the GLMM regression are summarized in Table 3. None of the fixed predictors cumulative experience group, sonographer ultrasound experience (years), cadaver age, cadaver BMI, or cadaver neck circumference were significantly associated with correct classification (*P* > 0.05 for all). The variance components for reader and cadaver were estimated at the boundary (< 0.001; singular fit), suggesting minimal residual between-cluster variability after accounting for fixed effects; these variance estimates should be interpreted cautiously given the small number of cadavers (*n* = 3).

**Table 3:** Mixed-effects logistic regression for correct classification

Random effect	SD	Variance		
Random-effects variance components				
Sonographer	<0.001	<0.001		
Cadaver	<0.001	<0.001		

Predictor	Category / Unit	OR	95% CI	p-value
Cumulative experience group	Reference = Developer	—	—	—
	1–5 scans	0.48	0.09 – 2.44	0.37
	6–10 scans	0.59	0.11 – 3.11	0.53
	11–15 scans	0.48	0.07 – 3.22	0.45
	16–20 scans	0.66	0.09 – 4.76	0.68
	21–25 scans	0.82	0.17 – 3.90	0.80
	26–30 scans	1.42	0.26 – 7.93	0.69
Sonographer experience	per year	0.68	0.43 – 1.09	0.11
Cadaver age	per year	1.12	0.70 – 1.79	0.64
Cadaver BMI	per kg/m²	0.74	0.32 – 1.68	0.47
Cadaver neck circumference	per cm	0.95	0.86-1.06	0.37

Generalized linear mixed model with binomial logit link; crossed random intercepts for sonographer and cadaver (n = 390 assessments; 12 readers; 3 cadavers). Fixed effects included cumulative experience (Developer reference; 1–5, 6–10, 11–15, 16–20, 21–25, 26–30 scans), sonographer ultrasound experience (years), cadaver age (years), cadaver BMI (kg/m²) and cadaver neck circumference (cm). Odds ratios (OR) are shown with two-sided Wald 95% confidence intervals and corresponding p-values. Random-effect variances for sonographer and cadaver were estimated at the boundary (<0.001; singular fit), which can occur with a small number of clusters (particularly cadavers, n=3) and limited residual heterogeneity; these variance estimates should therefore be interpreted cautiously. An OR > 1 indicates higher odds of correct classification

## Discussion

In this diagnostic accuracy study, the double twist sign showed high overall performance (success 90.6%) with rapid classification (mean = 18 s). Detection of deep placement was highly accurate (sensitivity and specificity ≈ 99%) with a mean time = 6 s.

The time to identify tube position was similar as to previous research (Gottlieb 2022) and is much faster than acquiring a chest X-ray. Identifying superficial tube position required more practice than detecting deep tubes, which was reflected in the lower overall sensitivity for superficial placements (82.1%; 95% CI 74.1–88.0). However, even for superficial tubes the double twist sign maintained very high specificity (95.5%; 95% CI 93.5–96.9). As shown in Fig. 2, sensitivity for superficial positions increased during the initial experience bins and then plateaued, while specificity showed a similar pattern (Figure S3). This suggests that, after a relatively short learning phase, clinicians can apply the double twist sign reliably in routine practice, including for superficial tube positions. These test characteristics are similar or better as compared to previous research in which a saline-filled cuff was used [12, 13]. Studies performed by Gottlieb showed discrimination between correct and deep tube position with a sensitivity of 99% and specificity of 97% [13]. Accuracy of ultrasound to discriminate between correct, deep and superficial tube position was 85% [12]. In another study in patients undergoing anesthesia and intubation with a saline-filled cuff, ultrasound was 100% sensitive and specific to detect superficial positions [14].

Skills acquired on one cadaver appeared transferable to others with differing anthropometric characteristics, such as higher BMI or neck circumference. Performance did not significantly differ between residents and intensivists, nor was it influenced by years of ultrasound experience or reader age. The mixed-effects analysis showed no detectable learning effect beyond the initial cases, suggesting that the double twist sign is a rapid and reliable method for estimating tracheal tube depth. It demonstrated particularly high accuracy in identifying and excluding deep tube positions and maintained good sensitivity for detecting superficial placements after limited practice. To our knowledge, this is the first study in which ultrasound reliably detected tube depth, discriminating between adequate, deep and superficial positions with a cuff inflated with air. In contrast to previous research on saline-filled cuffs, this technique can be directly applied without the need to change intubation procedure or need for deflation and inflation of the cuff. The latter was investigated by Patil et al., by temporarily replacing the cuff with saline and repositioning all tubes by placing the upper end of cuff at the level of the 3rd or 4th tracheal ring [15]. This resulted in adequate tube depth in 95% of patients but required repositioning of all tubes requiring potentially unnecessary repeated deflation and inflation of the cuff [15].

Comparison with current standard of care. In current practice, tracheal placement is typically confirmed with continuous waveform capnography, while tube depth is commonly assessed using centimeter markings, auscultation, and chest radiography. However, auscultation and tube markings are imperfect for depth assessment, and radiography can be delayed or unavailable in some settings. The double twist sign is not proposed to replace capnography for confirming tracheal placement; rather, it may provide rapid bedside information about tube depth particularly to identify potentially harmful deep placement while awaiting or complementing standard confirmation by chest radiography.

Major strengths of this study were the use of air-filled cuffs, high accuracy and quick identification of tube depth. We developed an easy to learn two-step protocol with flowchart, which can be taught to novice sonographers within 15 min. In contrast to previous research, there was no need to count tracheal rings [12, 15, 19]. As the ultrasound at the cricothyroid membrane was only evaluated if the double twist sign was positive in the suprasternal notch, a deep position could be identified in a matter of seconds. Importantly, this study showed that the double twist can be evaluated with a handheld ultrasound device, allowing it to be applied in pre-hospital and low resource settings. Given its high detection of deep malposition, speed (seconds rather than minutes), single-operator workflow, and absence of radiation, the double twist sign sign may serve as a bedside adjunct for early suspicion of deep placement while awaiting standard confirmation. Chest radiography remains the standard method for confirming tube depth in many institutions, and the diagnostic performance of this technique in live ventilated patients, including those with obesity, anterior neck edema, cervical immobilization, or significant motion must be established before changes to clinical workflows can be recommended.

Limitations and external validity. First, this was an embalmed cadaver study with only three cadavers, limiting generalizability across the range of airway anatomy encountered in clinical practice. Embalming and post-mortem changes (altered tissue compliance and stiffness, absent perfusion, and absence of muscle tone) may modify how rotational motion is transmitted and visualized, potentially changing the appearance or detectability of the twist signal compared with living patients. Second, our model did not reproduce dynamic clinical conditions such as spontaneous breathing, mechanical ventilation–related cephalad/caudal tube excursion, coughing, patient movement, or changes in neck position, all of which may influence test performance. Third, challenging clinical contexts (e.g., obesity, anterior neck tissue edema, subcutaneous emphysema, or cervical immobilization with collars) were not represented and may reduce image quality and/or maneuver feasibility. Fourth, two ‘expert’ operators were technique developers and study authors, which may overestimate performance due to familiarity and expectation bias. Independent validation by non-developer operators and multicenter clinical studies are therefore required.

Moreover, the cadaver model did not allow to evaluate for potential damage. However, it was considered unlikely for the double twist to be cause any damage to the trachea, as a similar movement of the tube occurs if the head of a patient is rotated sideways. The small number of cadavers (*n* = 3) limits precision of between-cadaver variance estimates and generalizability.

An additional limitation is that novice users practiced on cadaver A and then proceeded in a fixed order (A to B to C). This design may introduce learning-curve and order effects that cannot be fully separated from cadaver-specific sonographic difficulty. Therefore, statements regarding transferability across anatomies should be interpreted cautiously and should be reassessed in clinical studies with broader anatomic variation and randomized case order where feasible.

Future studies should prospectively evaluate feasibility and accuracy during ongoing ventilation in ICU and operating room conditions, including coughing, head and neck repositioning, and different ventilator settings. Studies should include patients with obesity, anterior neck edema, and cervical immobilization, and quantify interrater reliability across operators. A pragmatic design comparing time-to-recognition of malposition and diagnostic accuracy against chest radiography (and bronchoscopy when clinically indicated) would clarify how the double twist sign can be integrated as an adjunct into airway confirmation workflows.

## Conclusion

In an embalmed cadaver model, the double twist sign enabled rapid ultrasound-based classification of endotracheal tube depth with high diagnostic accuracy, particularly for deep placement, using standard air-filled cuffs. These findings support feasibility, but clinical validation in live ventilated patients—including comparison with chest radiography and evaluation in challenging anatomies and dynamic conditions—is required before clinical implementation recommendations can be made.

### Take-home message

Addressing the issue of malpositioned tubes and associated patient harm, the double twist sign was evaluated as a novel ultrasound tool to estimate endotracheal tube depth in a cadaver model. The test showed to reliably and quickly assess tube depth using standard air-inflated cuffs andcould be taught to novice users in 15 min. Because cadaver conditions differ from live ventilated patients, prospective clinical validation (including comparison with chest radiography) is required.

## Supplementary Information

Supplementary Material 1: Video S1 – Twist sign at the suprasternal notch. Transverse neck ultrasound at the suprasternal notch during deliberate endotracheal tube (ETT) rotation (corner-to-corner) in the mouth. A positive twist sign is demonstrated as localized peri-tracheal rotational tissue motion at the scanning level, synchronous with tube rotation. In the double twist framework, a negative suprasternal-notch twist sign indicates deep tube placement (cuff below the suprasternal notch), whereas a positive finding prompts the second step at the cricothyroid membrane. The clip is shown in real time

Supplementary Material 2: Video S2 – Twist sign at the cricothyroid membrane. Transverse neck ultrasound at the cricothyroid membrane during deliberate ETT rotation (corner-to-corner) in the mouth. A positive twist sign is shown as peri-tracheal rotational tissue motion at this level. In the double twist framework, when the suprasternal-notch assessment is positive, a negative cricothyroid-membrane twist sign is consistent with adequate depth, whereas a positive finding at both landmarks indicates superficial placement. The clip is shown in real time.

Supplementary material 3.

## Data Availability

The de-identified dataset supporting the conclusions of this article is available from the corresponding author on reasonable request.

## Abbreviations

ALIFE: Amsterdam Leiden IC focused echography
BMI: Body mass index
CI: Confidence interval
DEUS: Dutch emergency ultrasound
EDC: Electronic data capture
ETT: Endotracheal tube
F4L: Fix for Life (cadaver model)
FATE: Focus-assessed transthoracic echocardiography
GEE: Generalized estimating equations
GLMM: Generalized linear mixed model
ICARUS: Intensive care ultrasound
ICU: Intensive care unit
LUMC: Leiden university medical center
NPV: Negative predictive value
NIV: Dutch internist association
NVIC: Dutch association for intensive care
OR: Odds ratio
POCUS: Point-of-care ultrasound
PPV: Positive predictive value
SD: Standard deviation
SLICE: Shocked/Short of breath lungs IVC cardiac and extra regions as indicated
SPSS: Statistical package for the social sciences
TEE: Transoesophageal ultrasound
TTE: Transthoracic echocardiography
WINFOCUS: World interactive network focused on critical ultrasound
