# Supplementary material for: In-depth tube assessment by ultrasound: double twist sign to evaluate endotracheal tube depth—results of a cadaver diagnostic accuracy study
Source: Intensive Care Med Exp. 2026 Jul 17;14:93. doi: 10.1186/s40635-026-00937-x (PMC13379563; doi:10.1186/s40635-026-00937-x)
Supplement: Supplementary file 3 — Supplementary material 3. [file 40635_2026_937_MOESM3_ESM.docx]

**Supplemental data**

**Supplemental Figure S1**

Cadaver selection


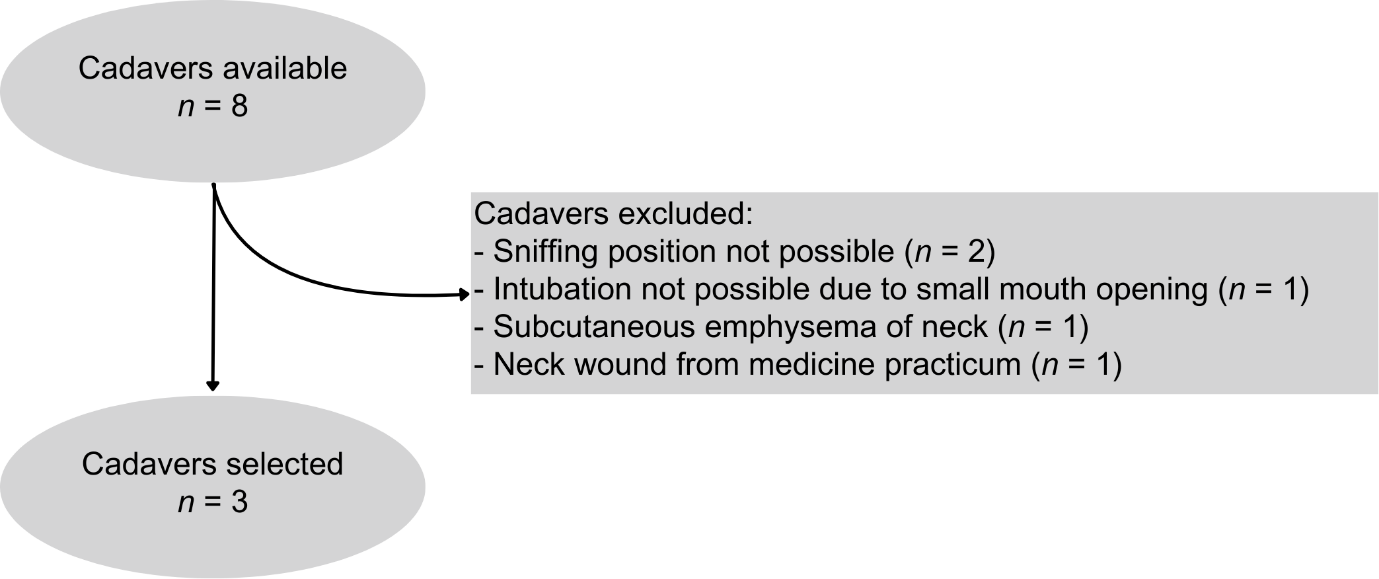


**Supplemental Figure S2**

Teaching material for the double twist sign


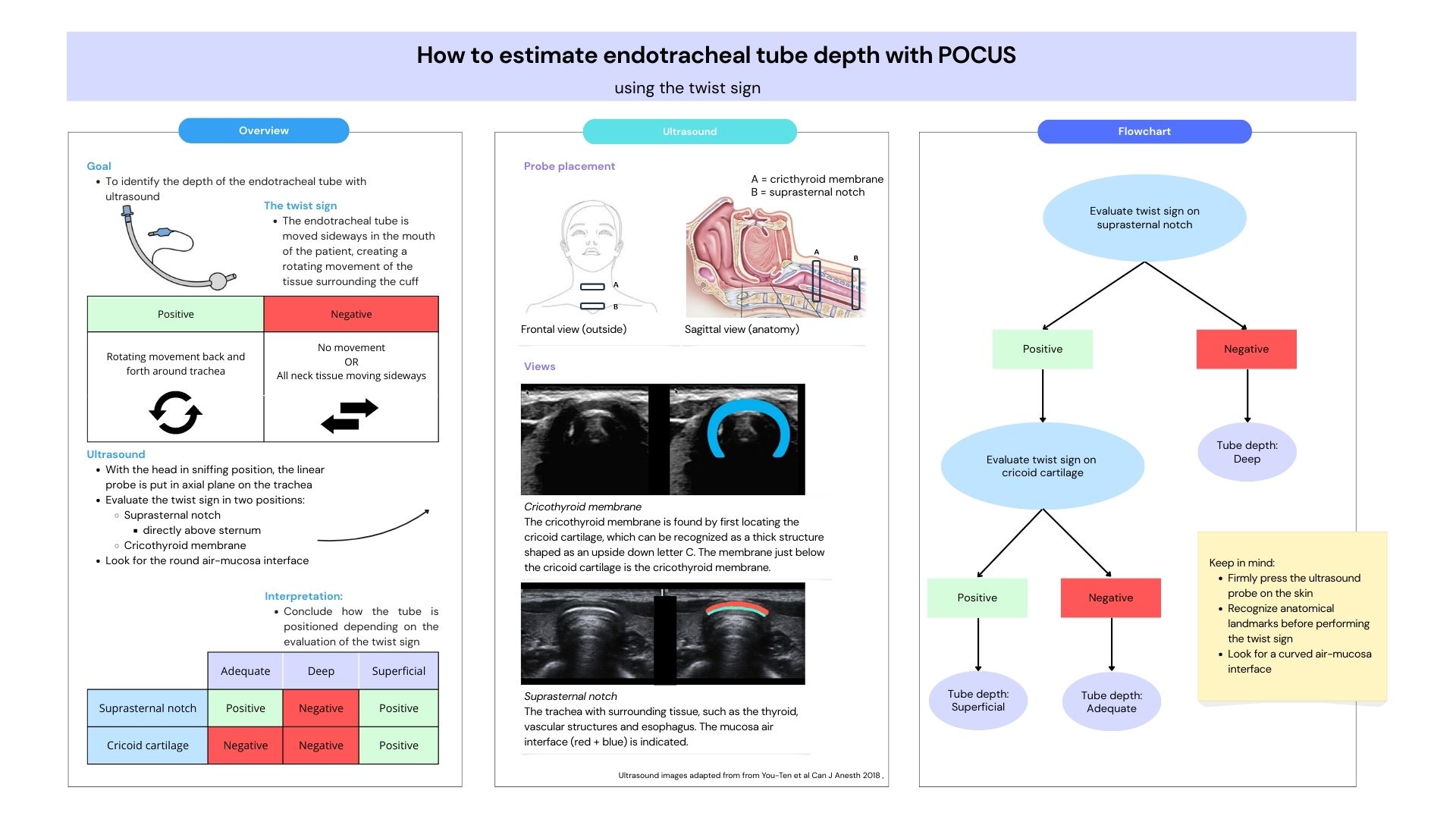


**Supplemental Figure S3: Clustered specificity learning curves for deep and superficial tube position detection by cumulative ultrasound experience.**

Specificity (points) with 95% confidence intervals (bars) for deep and superficial tube positions across cumulative ultrasound experience (grouped in blocks of 5 scans). Estimates are from a mixed‑effects logistic regression with random intercepts for sonographer and cadaver***.***


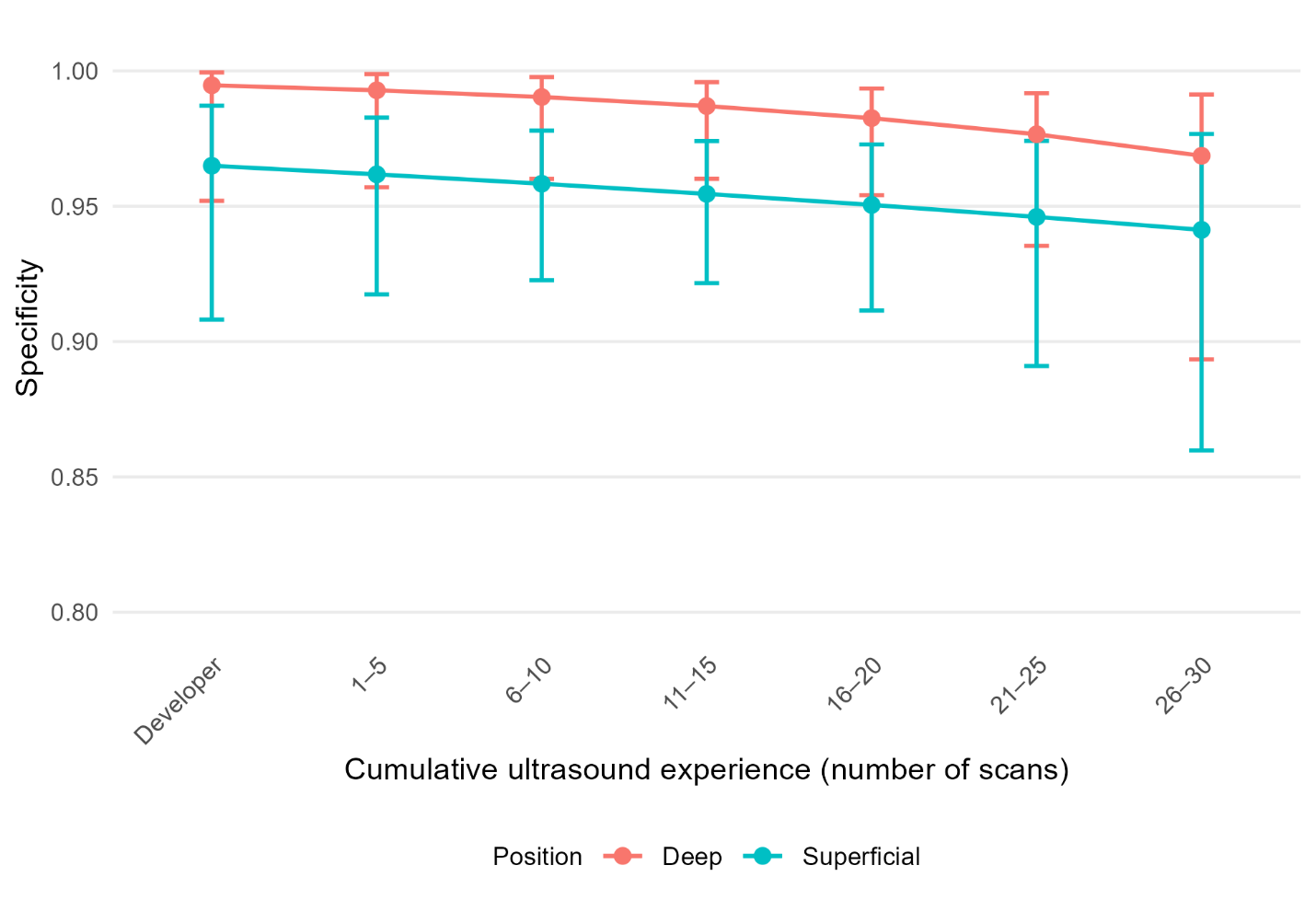


**Supplemental Table S1**

Cadaveric features

| Cadaver | Age at time of death (years) | Gender | Length (m) | Weight (kg) | BMI (kg/m2) | Neck circum-ference * (cm) | Neck length ** (cm) | Trachea length *** (cm) |
| --- | --- | --- | --- | --- | --- | --- | --- | --- |
| A | 83 | Female | 1.68 | 62 | 22 | 38 | 8 | 11 |
| B | 87 | Female | 1.66 | 61 | 22 | 47 | 12 | 13 |
| C | 91 | Male | 1.86 | 91 | 26 | 56 | 14 | 14 |

* Neck circumference (cm) was measured using non-stretchable plastic tape to the nearest 1 cm and was measured from the level just below the laryngeal prominence perpendicular to the long axis of the neck

** Neck length (cm) was defined as the sternomental distance, measured using non-stretchable plastic tape to the nearest 1 cm with the head positioned in sniffing position

*** Trachea length (cm) is defined as the distance between the vocal cords and the internal ridge of the main carina, measured using the depth markings on the tube

BMI: Body mass index

**Supplemental Table S2**

Sonographer information

| Sonographer | Developer | Role | Age (years) | Ultrasound courses | Experience with ultrasound (years) | Experience with ultrasound of airway (yes/no) |
| --- | --- | --- | --- | --- | --- | --- |
| 1 | yes | Intensivist | 48 | Basic ultrasound (NVIC) WINFOCUS  SLICE  TTE | 20 | yes |
| 2 | yes | Resident | 33 | Basic ultrasound (NIV) | 3 | yes |
| 3 | no | Intensivist | 40 | Lung ultrasound FATE TTE Abdominal ultrasound | 8 | yes |
| 4 | no | Intensivist | 59 | Lung ultrasound TTE TEE | 8 | no |
| 5 | no | Intensivist | 60 | WINFOCUS  SLICE TTE | 9 | no |
| 6 | no | Intensivist | 55 | Basic ultrasound (NVIC) | 12 | yes |
| 7 | no | Intensivist | 50 | Basic ultrasound (LIFE) Advanced ultrasound (ALIFE) SLICE  ABCD | 10 | yes |
| 8 | no | Resident | 29 | Pain block ultrasound | 4 | yes |
| 9 | no | Resident | 31 | None | 1 | no |
| 10 | no | Resident | 39 | Basic ultrasound (NIV) | 4 | yes |
| 11 | no | Resident | 38 | Basic ultrasound (DEUS) | 6 | no |
| 12 | no | Resident | 29 | Basic ultrasound (ICARUS) | 2 | no |

ALIFE: Amsterdam Leiden IC focused echography, DEUS: Dutch Emergency Ultrasound, FATE: Focus-Assessed Transthoracic Echocardiography, ICARUS: Intensive Care Ultrasound, SLICE: Shocked/Short of breath Lungs IVC Cardiac and Extra regions as indicated, TTE: Transthoracic echocardiography, TEE: Transoesophageal ultrasound, NIV: Dutch Internist Association, NVIC: Dutch Association for Intensive Care, WINFOCUS: World Interactive Network Focused on Critical Ultrasound
